# Alkane degradation under anoxic conditions by a nitrate-reducing bacterium with possible involvement of the electron acceptor in substrate activation

**DOI:** 10.1111/j.1758-2229.2010.00198.x

**Published:** 2011-02

**Authors:** Johannes Zedelius, Ralf Rabus, Olav Grundmann, Insa Werner, Danny Brodkorb, Frank Schreiber, Petra Ehrenreich, Astrid Behrends, Heinz Wilkes, Michael Kube, Richard Reinhardt, Friedrich Widdel

**Affiliations:** 1 Max-Planck-Institut für Marine MikrobiologieCelsiusstraße 1, 28359 Bremen, Germany; 2 Institut für Chemie und Biologie des Meeres, Universität OldenburgCarl-von-Ossietzky Str. 9-11, 26111 Oldenburg, Germany; 3 GeoForschungsZentrum PotsdamTelegrafenberg, 14473 Potsdam, Germany; 4 Max-Planck-Institut für Molekulare GenetikIhnestraße 73, 14195 Berlin, Germany; 5 Max-Planck-Institut für PflanzenzüchtungsforschungCarl-von-Linné-Weg 10, 50829 Köln, Germany

## Abstract

Microorganisms can degrade saturated hydrocarbons (alkanes) not only under oxic but also under anoxic conditions. Three denitrifying isolates (strains HxN1, OcN1, HdN1) able to grow under anoxic conditions by coupling alkane oxidation to CO_2_ with NO_3_^−^ reduction to N_2_ were compared with respect to their alkane metabolism. Strains HxN1 and OcN1, which are both *Betaproteobacteria*, utilized *n*-alkanes from C_6_ to C_8_ and C_8_ to C_12_ respectively. Both activate alkanes anaerobically in a fumarate-dependent reaction yielding alkylsuccinates, as suggested by present and previous metabolite and gene analyses. However, strain HdN1 was unique in several respects. It belongs to the *Gammaproteobacteria* and was more versatile towards alkanes, utilizing the range from C_6_ to C_30_. Neither analysis of metabolites nor analysis of genes in the complete genome sequence of strain HdN1 hinted at fumarate-dependent alkane activation. Moreover, whereas strains HxN1 and OcN1 grew with alkanes and NO_3_^−^, NO_2_^−^ or N_2_O added to the medium, strain HdN1 oxidized alkanes only with NO_3_^−^ or NO_2_^−^ but not with added N_2_O; but N_2_O was readily used for growth with long-chain alcohols or fatty acids. Results suggest that NO_2_^−^ or a subsequently formed nitrogen compound other than N_2_O is needed for alkane activation in strain HdN1. From an energetic point of view, nitrogen–oxygen species are generally rather strong oxidants. They may enable enzymatic mechanisms that are not possible under conditions of sulfate reduction or methanogenesis and thus allow a special mode of alkane activation.

## Introduction

Saturated hydrocarbons (alkanes) as major constituents of petroleum (Tissot and Welte, 1984) enter the environment via natural seeps or accidental spills, or due to the use of refined petroleum products. Furthermore, alkanes are widespread products of living organisms (Birch and Bachofen, 1988). Aerobic alkane biodegradation, in particular the initial O_2_-dependent activation by monooxygenases, has been studied since many decades (Rojo, 2009). In recent years, alkanes were also shown to be degraded anaerobically with nitrate (Ehrenreich *et al*., 2000; Bonin *et al*., 2004; Grossi *et al*., 2008; Callaghan *et al*., 2009) or sulfate (Aeckersberg *et al*., 1991; 1998; So and Young, 1999; Cravo-Laureau *et al*., 2004; Davidova *et al*., 2006; Kniemeyer *et al*., 2007; Higashioka *et al*., 2009) as electron acceptor, or under conditions of methanogenesis (Zengler *et al*., 1999; Anderson and Lovley, 2000; Jones *et al*., 2008). The only established mechanism for anaerobic activation of alkanes to date is the radical-catalysed addition to fumarate yielding alkylsuccinates (Kropp *et al*., 2000; Rabus *et al*., 2001; Heider, 2007). Genes [designated *mas*, for (1-methylalkyl)succinate synthase; or *ass*, for alkylsuccinate synthase] encoding the putative enzyme have been detected in a nitrate-reducing (Grundmann *et al*., 2008) and a sulfate-reducing (Callaghan *et al*., 2008) strain. Still, an alternative possibility for anaerobic alkane activation has been suggested on the basis of cell fatty acid and isotope labelling analysis (Aeckersberg *et al*., 1998; So *et al*., 2003; Callaghan *et al*., 2006).

Of three denitrifying strains, HxN1, OcN1 and HdN1, that were isolated with *n*-hexane, *n*-octane and*n*-hexadecane, respectively (Ehrenreich *et al*., 2000), only the first one has been formerly studied with respect to its alkane metabolism (Rabus *et al*., 2001; Wilkes *et al*., 2002; Grundmann *et al*., 2008). A subsequent comparative study including the two other strains revealed that also strain OcN1 formed alkylsuccinates during growth with alkanes and harboured a gene apparently encoding the responsible enzyme. In contrast, alkylsuccinates were not detectable in strain HdN1, and its complete genome sequence did not reveal any gene likely to encode (1-methylalkyl)succinate or alkylsuccinate synthase. A unique physiological characteristic of strain HdN1 was that it did not grow with alkanes if N_2_O was added instead of NO_3_^−^, whereas growth with alcohols and fatty acids readily occurred with N_2_O. In contrast, strains HxN1 and OcN1 grew well with N_2_O and alkanes. These findings suggest that alkane activation in strain HdN1 differs principally from alkane activation in strains HxN1 and OcN1 and requires an NO_3_^−^-derived compound other than N_2_O.

## Results and discussion

### Cultivation, phylogenetic relationships, morphology and purity control

The focus of this study is on strain HdN1 and its apparently unusual physiology with respect to *n*-alkane utilization. The isolation of strain HdN1 along with that of strains OcN1 and HxN1, a few substrate tests, and the capacity for complete alkane oxidation in anoxic medium with NO_3_^−^ have been documented previously (Ehrenreich *et al*., 2000). Unless indicated otherwise, the strains were grown in conventional HCO_3_^−^/CO_2_-buffered defined medium (Rabus and Widdel, 1995) with alkanes as the only organic substrates.

The study of anaerobic microbial hydrocarbon utilization requires the strict exclusion of any traces of O_2_ from air which through monooxygenases could lead to hydroxyl compounds (which can be further degraded anaerobically). Hence, in addition to physical exclusion of air (Widdel and Bak, 1992), the presence of a reductant (‘redox buffer’) is advisable. Unlike sulfate-reducing bacteria that form a chemical reducing agent, sulfide, nitrate-reducing bacteria do not produce a reductant. Addition of sulfide (or other reducing sulfur compounds) is inappropriate because it is easily oxidized in by-reactions of the ‘high-potential’ nitrate reduction pathway, or because it can inhibit denitrifiers (F. Widdel, unpubl. results). We therefore added ascorbate (4 mM) as a mild reductant (Rabus and Widdel, 1995; Ehrenreich *et al*., 2000; Widdel, 2009). Ascorbate did not serve as substrate for growth and nitrate reduction, as revealed in control incubations with ascorbate alone. We also verified that ascorbate in our medium did not scavenge nitrite, the intermediate of nitrate reduction, by chemical reaction. If sterile medium with ascorbate (pH 7.2) and NaNO_2_ (2 mM) was incubated for 10 days and analysed by ion chromatography (Rabus and Widdel, 1995), there was no noticeable decrease of the nitrite concentration. At low pH, reduction of the protonated form (HNO_2_) by ascorbic acid to yield nitric oxide can be significant (Yamasaki, 2000). Furthermore, tests were carried out to exclude an adverse physiological effect of ascorbate. Strain HdN1 was grown with *n*-tetradecane and NO_3_^−^ or NO_2_^−^ in ascorbate-containing medium as well as in ascorbate-free medium deoxygenated by vigorous sparging with N_2_. The cultures with and without ascorbate grew equally well.

**Fig. 1:**
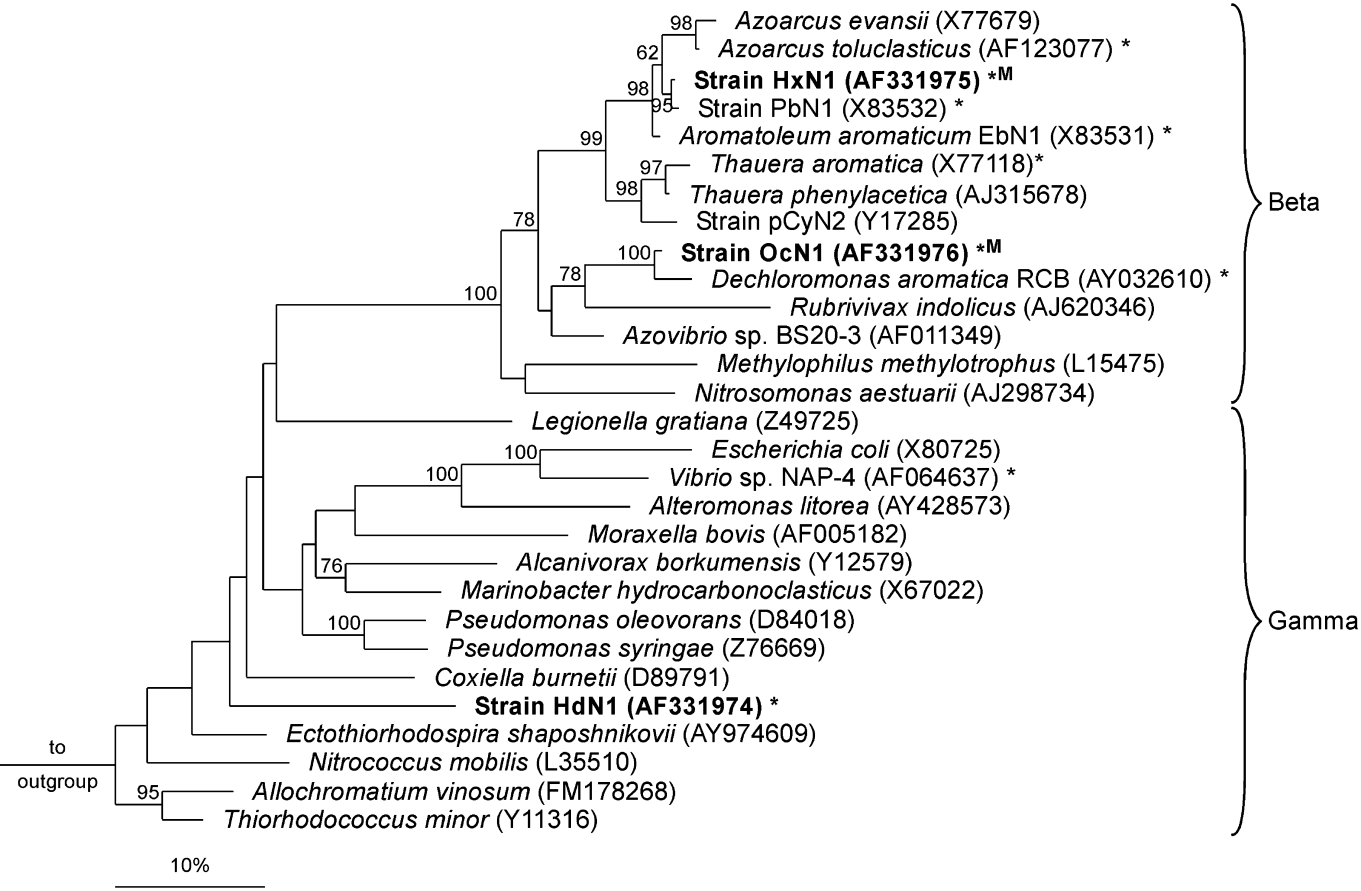
Phylogenetic (16S rRNA-based) affiliation of strain HdN1 with selected *Beta*- and *Gammaproteobacteria* including other strains able to degrade aromatic or saturated petroleum hydrocarbons with nitrate (*). Strains able to degrade *n*-alkanes anaerobically are highlighted in bold; occurrence of (1-methylalkyl)succinate formation for alkane activation is also indicated (^M^). Bootstrap values (%; only > 60% shown) were obtained after 1000 resamplings. Scale bar, 10% estimated sequence divergence.

Strain HdN1 affiliates with the *Gammaproteobacteria*, whereas strain OcN1 and HxN1 are members of the *Betaproteobacteria* (Fig. 1). Most nitrate-reducing bacteria enriched and isolated with various aromatic or saturated petroleum hydrocarbons are *Betaproteobacteria* (Widdel *et al*., 2009). Some denitrifying strains that degrade petroleum hydrocarbons are *Gammaproteobacteria*; these also include alkane degraders affiliating with *Marinobacter* sp. (Bonin *et al*., 2004) and *Pseudomonas balearica* (Grossi *et al*., 2008).

**Fig. 2:**
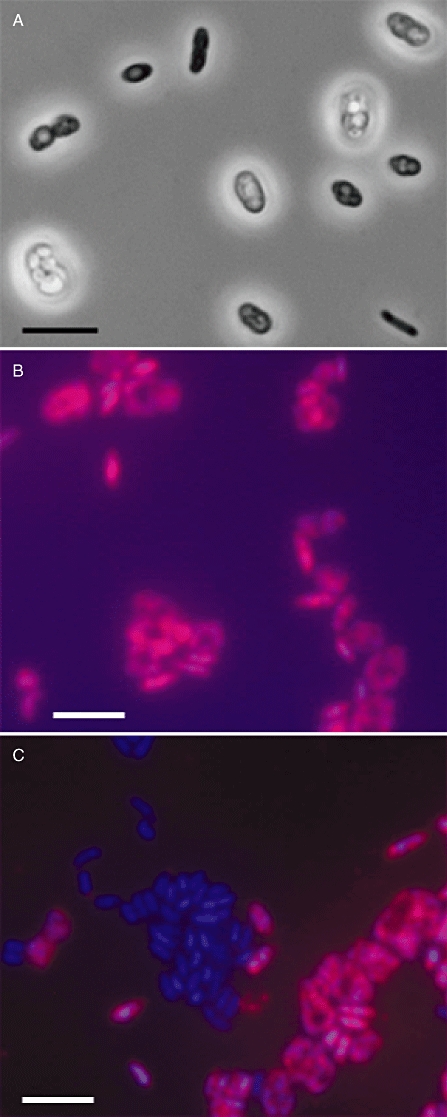
Microscopic images of strain HdN1. A. Highly variable cell forms of strain HdN1 grown anaerobically with hexadecane and nitrate. Phase-contrast micrographs of viable cells. Bar, 5 µm. B. Cells from a pure culture of strain HdN1 hybridized with a specific 16S rRNA-targeted oligonucleotide probe and stained with DAPI. The image represents an overlay of the probe and the DAPI signal. Bar, 5 µm. C. Mixed cells of strains HdN1 and OcN1 hybridized, stained and visualized as in (B). Bar, 5 µm.

The cell shape of strain HdN1 was unusually variable and significantly influenced by the organic growth substrate (Ehrenreich *et al*., 2000). In particular long-chain alkanes caused swelling of a large fraction of the cells. In such cells, spacious inclusions resembling storage compounds could be seen at high magnification (Fig. 2A). However, polyhydroxyalkanoates were not detectable (A. Steinbüchel, pers. comm.) by gas chromatography following acidic hydrolysis and methylation of freeze-dried cells (Steinbüchel and Wiese, 1992). Cells in alkane cultures tended to grow in close contact with the overlying insoluble hydrocarbon phase. The bulk of alkane-grown cells was buoyant, possibly due to association with or storage of alkane droplets. Alkane storage and buoyancy is a phenomenon known from aerobic alkane degraders (Scott and Finnerty, 1976). This behaviour rendered harvesting by centrifugation difficult. A minor fraction of the cells was motile.

Thorough purity tests excluded that cell shape heterogeneity in cultures of strain HdN1 was due to accompanying microorganisms. First, repeated aerobic and anaerobic (with NO_3_^−^) liquid dilution series (according to the most probable number technique) were carried out separately with *n*-tetradecane or *n*-valerate (*n*-pentanoate). All cultures derived from the highest positive dilution tubes were microscopically indistinguishable and always able to use both, tetradecane and valerate. Second, cultures were streaked on agar plates containing valerate and yeast extract and incubated in a jar under air with 3% CO_2_. All well-separated valerate-grown colonies transferred to anoxic liquid media grew again with tetradecane, and cultures had the microscopic appearance as before. Third, strain HdN1 was mixed with strain OcN1, and a specific 16S rRNA-targeting fluorescent oligonucleotide probe (Appendix S1) was applied. Whereas in the pure culture all cells exhibited the specific hybridization signal (Fig. 2B), the mixed culture contained in addition the expected non-hybridizing cells that exhibited only the general fluorescent stain (Fig. 2C). Hence, strain HdN1 is in principle distinguishable from contaminants by specific probing.

### Anaerobic growth tests with alkanes and alkanoates

The capability of strain HdN1 for complete hexadecane oxidation with nitrate according to 5 C_16_H_34_ + 98 NO_3_^−^ + 18 H^+^→ 80 HCO_3_^−^ + 49 N_2_ + 54 H_2_O has been verified formerly with small, precisely quantifiable amounts of alkane (Ehrenreich *et al*., 2000). In all subsequent experiments, significantly higher amounts of alkanes were added than could be oxidized by the electron acceptor (10 mM NO_3_^−^). In this way, a large contact area between the insoluble hydrocarbon and the aqueous phase was provided which favoured growth (Widdel, 2009). In further growth tests, alkanes with carbon chains ≤ C_10_ were provided as solutions in 2,2,4,4,6,8,8-heptamethylnonane (HMN) as an inert carrier phase to avoid toxic effects (Appendix S1). Tests revealed that strain HdN1 utilized *n*-alkanes from C_6_ (*n*-hexane) to C_30_ (*n*-triacontane) as carbon sources and electron donors (C_6_ to C_20_, C_24_, C_26_, C_28_, C_30_, C_36_ and C_40_ tested). Fastest growth was observed in the range from C_14_ (tetradecane) to C_18_ (octadecane). With an inoculum size of 1% (v/v), full growth and complete NO_3_^−^ consumption occurred within 7 days. A doubling time of 11–13 h during early growth was estimated from an analysis of the nitrate consumption curve (see also Ehrenreich *et al*., 2000). (Inhomogeneous growth and alkane droplets prevented measurement of the optical density as a growth parameter.) Growth with alkanes of shorter or longer chains was slower (two- to threefold time required for full growth and NO_3_^−^ consumption). The other strains, HxN1 and OcN1, utilized a significantly narrower range of alkanes, which was from C_6_ to C_8_ (*n*-octane) and C_8_ to C_12_ (*n*-dodecane) respectively. Also alkane-utilizing sulfate-reducing bacteria utilized a narrower range (Rueter *et al*., 1994; Aeckersberg *et al*., 1998).

Strain HdN1 utilized monocarboxylic acids (sodium salts; method of preparation and addition given by Widdel and Bak, 1992; see also Appendix S1) from acetate to stearate (C_2_–C_18_; higher fatty acids not tested), with best growth (roughly twice as fast as with alkanes) with valerate (C_5_) and with fatty acids from *n*-decanoate (C_10_) to stearate. Some primary linear alcohols were also tested (C_8_ and C_10_ provided as solutions in HMN; C_14_ and C_16_ added as solid compounds). Strain HdN1 grew well with 1-decanol, 1-tetradecanol and 1-hexadecanol; growth with 1-octanol was poor, and no growth occurred with ethanol.

### Growth tests with different electron acceptors

All three strains grew also aerobically with alkanes. Examination of strain HdN1 in more detail revealed that almost the same range of *n*-alkanes (and fatty acids) was oxidized with O_2_ as in anaerobic cultures with NO_3_^−^. Only *n*-hexane was not utilized so far with O_2_. Another slight difference between aerobic and anaerobic alkane utilization was observed if cultures grown with hexadecane were transferred to medium with tridecane (C_13_) or dodecane (C_12_). Whereas aerobic cultures grew immediately with the lighter alkanes, anaerobic cultures exhibited a lag-phase of > 10 days.

**Fig. 3:**
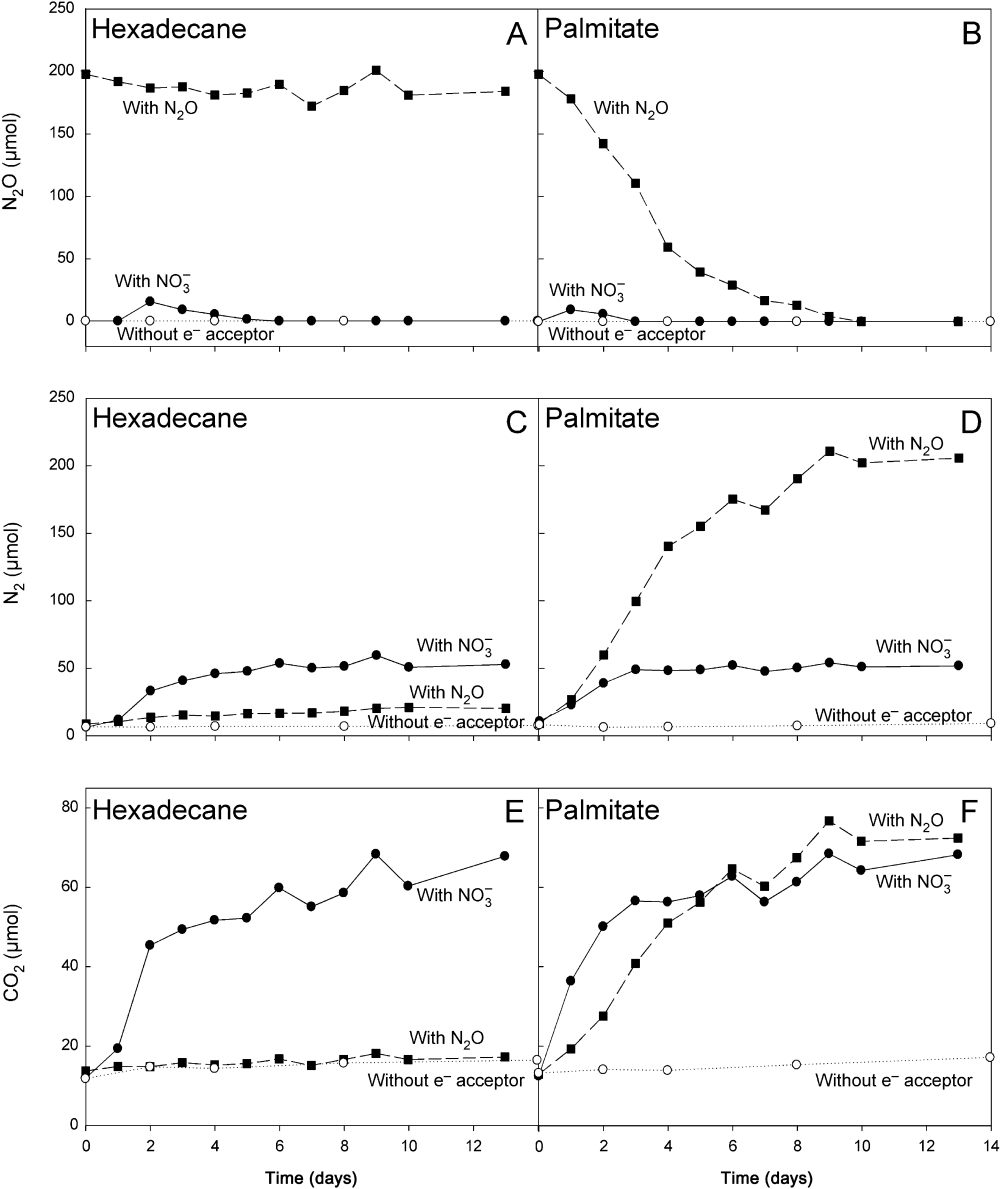
Time-courses of the formation of N_2_O (A and B), N_2_ (C and D) and CO_2_ (E and F) in anaerobic cultures of strain HdN1 with *n*-hexadecane (A, C and E) or palmitate (B, D and F). The electron acceptors were added in stoichiometrically limiting amounts (100 µmol of NO_3_^−^; *c*. 250 µmol of N_2_O) relative to the electron donor (171 µmol of hexadecane, advantage of large excess explained in text; 10 µmol of palmitate). Results show that alkane oxidation to CO_2_ was not possible with N_2_O, but readily occurred with NO_3_^−^. The functionalized compound, palmitate, was oxidized with N_2_O. Duplicates yielded the same results (not shown). Culture volumes of 10 ml (phosphate-buffered medium, pH ≈ 7.1, without addition of NaHCO_3_; Appendix S1) were incubated in 165 ml serum bottles under an argon headspace. N_2_O was injected as pure O_2_-free gas. Cultures were very gently shaken for a few minutes per day. Vigorous shaking had to be avoided because it impeded growth. Samples from the headspace were analysed with a gas chromatograph employing argon as carrier gas and a thermal conductivity detector. The calculated dissolved amounts of gases were added so as to obtain the total amounts in the bottles. Calculation was based on literature data (Wilhelm *et al*., 1977; Stumm and Morgan, 1995), assuming equilibrium (which may not have been fully reached due to limited agitation) and considering pH in the case of CO_2_.

The transient formation by strain HdN1 of NO_2_^−^ (≤ 1.5 mM; not shown) and N_2_O (Fig. 3A and B, lower curves) at low concentration during NO_3_^−^ reduction and the detection of N_2_ in all cultures grown under an argon atmosphere indicated the common denitrification pathway. To further examine the capability for efficient use of NO_2_^−^ and N_2_O, these electron acceptors were tested individually in the absence of NO_3_^−^.

Growth with alkanes also occurred with added NO_2_^−^ (instead of NO_3_^−^), but was slightly slowed down if more than 5 mM NO_2_^−^ was added. Furthermore, a lag-phase of *c*. 2 days was sometimes observed after inoculation of new medium with NO_2_^−^. Hence, several mM may be somewhat inhibitory.

**Fig. 4:**
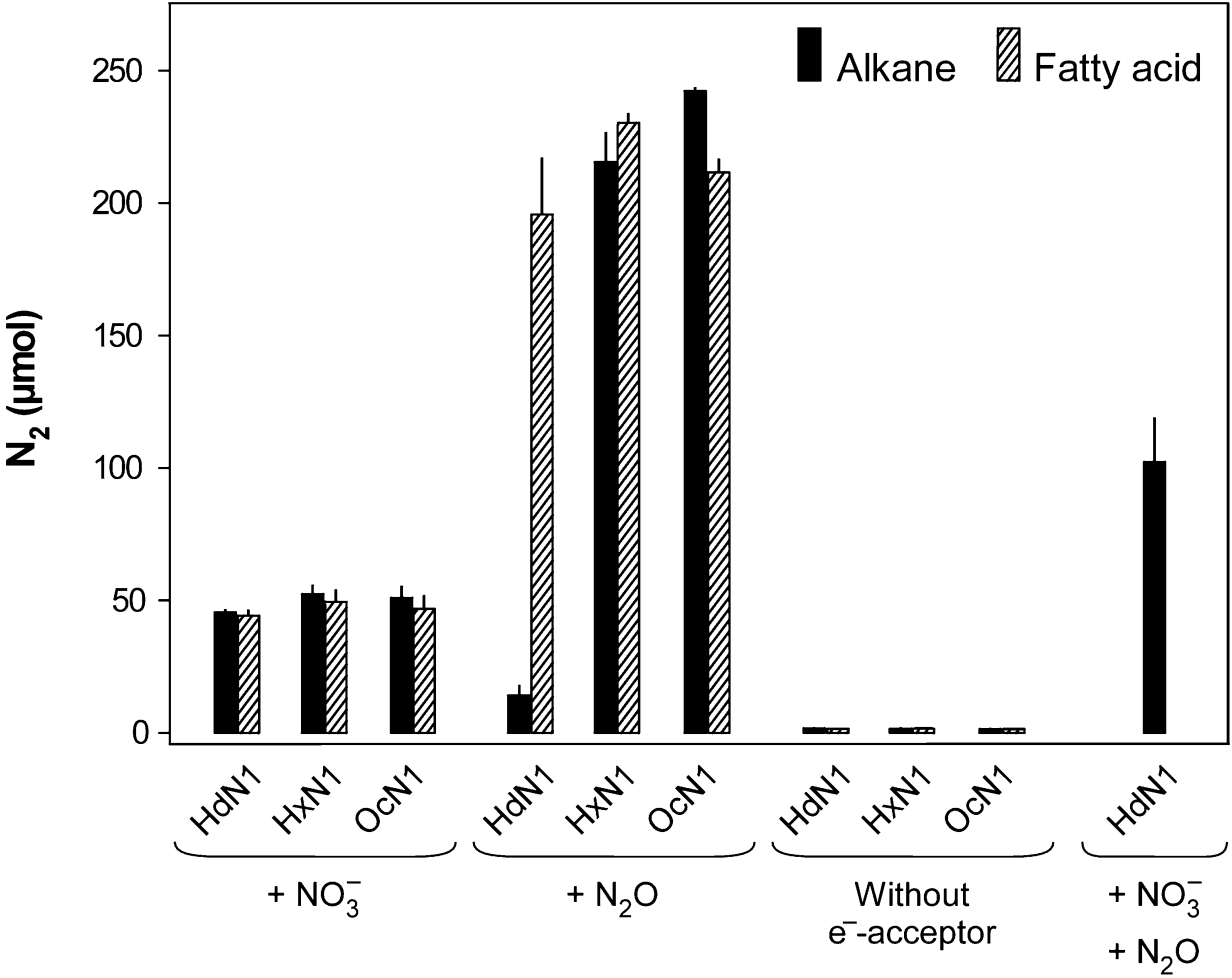
N_2_ formed in anaerobic cultures of strains HdN1, HxN1 and OcN1 with alkanes (black bars) or fatty acids (striated bars) and either NO_3_^−^ (100 µmol) or N_2_O (250 µmol). A control experiment with strain HdN1 for excluding N_2_O toxicity received both, NO_3_^−^ and N_2_O. Here, more N_2_ was formed than with NO_3_^−^ alone. This indicated that not only NO_3_^−^ but also N_2_O was used in the anaerobic respiratory chain if alkane degradation was enabled by NO_3_^−^. Data show that strain HdN1 could not use N_2_O alone for alkane degradation, in contrast to the other strains. Culture volumes of 10 ml were incubated in 20 ml butyl-rubber sealed tubes. Strain HdN1 received 171 µmol of pure *n*-hexadecane, or 10 µmol of palmitate. Strain HxN1 received 38 µmol of *n*-hexane (in 100 µl of heptamethylnonane as carrier), or 30 µmol of caproate. Strain OcN1 received 31 µmol of *n*-octane (in 100 µl of heptamethylnonane), or 30 µmol of caproate. Tubes were incubated nearly horizontally while contact of the hydrocarbon phase with the stopper was avoided (Widdel, 2009) as far as possible. Gas samples were withdrawn 11 days after inoculation and analysed (triplicates) as indicated in Fig. 3.

Surprisingly, strain HdN1 did not grow with alkanes in the growth tests with N_2_O. In accordance with the lack of growth, N_2_O was not consumed (Fig. 3A, upper curve), and N_2_ (Fig. 3C) or CO_2_ (Fig. 3E) were not formed. In contrast, growth with 1-tetradecanol, 1-hexadecanol or fatty acids was possible with added N_2_O, and consumption of N_2_O (Fig. 3B) as well as formation of N_2_ (Fig. 3D) and CO_2_ (Fig. 3F) was obvious. A minor formation of N_2_ from N_2_O during incubation with hexadecane can be explained by reduction with an endogenous electron source in the inoculum. The formation of N_2_ from N_2_O requires only 2 e^−^, whereas formation of N_2_ from NO_3_^−^ requires 10 e^−^ from an electron donor. The lack of alkane utilization with N_2_O was not due to specific inhibition. The same amount of N_2_O added to a culture with hexadecane and NO_3_^−^ did not inhibit growth. For physiological comparison, strains HxN1 and OcN1 were also incubated with N_2_O as the only electron acceptor and utilizable alkanes (*n*-hexane and *n*-octane respectively). These strains were able to grow with N_2_O and alkanes. Results are summarized in Fig. 4. The inability for coupling alkane utilization to N_2_O reduction is apparently unique for strain HdN1.

Other electron acceptors tested (concentrations in mM) but not utilized were sulfate (15), thiosulfate (5), sulfur (added as slurry), fumarate (10) and perchlorate (10). Toxic effects were excluded in controls containing in addition NO_3_^−^. In contrast, chlorate was toxic.

### Search for metabolites and genes involved in alkane degradation

Investigation of metabolites and genes involved in alkane degradation via addition to fumarate have been reported for strain HxN1 (Rabus *et al*., 2001; Wilkes *et al*., 2002; Grundmann *et al*., 2008). The presently performed metabolite analysis of strain OcN1 upon growth with *n*-octane and NO_3_^−^ revealed (1-methylheptyl)succinate (extraction, methylation and analysis as in Rabus *et al*., 2001; Wilkes *et al*., 2003), again indicating an activation via addition to fumarate. In contrast, alkyl-substituted succinates were never detectable in cultures and cells of strain HdN1. Another product searched for [by gas chromatography-mass spectrometry of extracts silylated with *N*,*O*-bis(trimethylsilyl)acetamide; Appendix S1] in anaerobic *n*-hexadecane cultures of strain HdN1 was 1-hexadecanol. If air was strictly excluded and if the culture was inactivated by heat (85°C; Rabus *et al*., 2001) before extraction, 1-hexadecanol was not detectable. In contrast, 1-hexadecanol was detected if the anaerobically grown culture was exposed to air for 20–30 min (data not shown). Such 1-alkanol formation is a long-known indicator of alkane monooxygenase activity (Britton, 1984). Metabolite analysis in anaerobic alkane degraders with facultative aerobic metabolism thus requires careful avoidance of artefacts due to reaction with O_2_ from air.

The gene possibly encoding the alkane-activating enzyme in strain OcN1 was retrieved via polymerase chain reaction with degenerate primers for *mas* and *ass* genes, generation of a probe and screening of a genomic library, similar as described for strain HxN1 (Grundmann *et al*., 2008). The derived amino acid sequence (Accession No. FN675935) revealed close relationships (Fig. S1) to the orthologue from strain HxN1 (Grundmann *et al*., 2008) and a sulfate-reducing bacterium (Callaghan *et al*., 2008). Attempts to amplify in an analogous manner *mas*- or *ass*-like genes from strain HdN1 failed. Therefore, a shotgun genomic library of strain HdN1 was established. This allowed assemblage of the complete genome sequence (4 587 455 bp; 3762 coding sequences; Accession No. FP929140; for some more details see Table S1). But neither this revealed *mas*- or *ass*-like genes (Table S2).

These findings suggested that the mechanism for alkane activation in strain HdN1, which has to involve the cleavage of a strong, apolar C−H bond, differs basically from the mechanism with fumarate as co-substrate in the two other strains.

### Linkage of alkane activation in strain HdN1 to the nitrate reduction pathway?

**Fig. 5:**
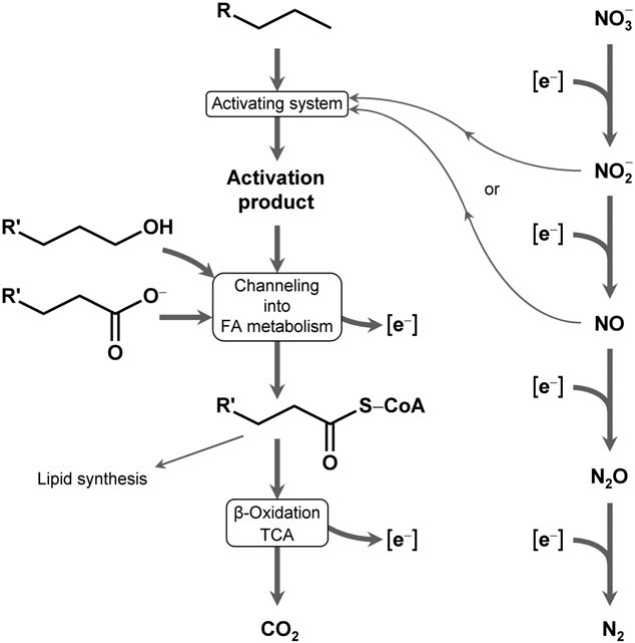
Hypothetical involvement of denitrification intermediates in alkane activation. The scheme offers an explanation for the inability of strain HdN1 to utilize *n*-alkanes with N_2_O alone (see Figs 3 and 4). It is assumed that a small proportion of NO_2_^−^ or NO is deviated from the respiratory chain for alkane activation. They may be used for activation indirectly (by yielding O_2_ that is used by alkane monooxygenase; or by giving rise to another reactive factor or enzyme centre) or directly (as co-reactants introducing a polar group). The alkyl residue R′ may or may not be identical with the original residue R (depending on the activation mechanism and alkane C-atom being attacked). FA, fatty acid; TCA, tricarboxylic acid cycle.

The distinctive results of the incubation experiments with either alkanes or functionalized (O-group-containing) substrates and N_2_O may offer a clue as to how strain HdN1 could initiate alkane degradation under anoxic conditions. The electron acceptor tests with functionalized electron donors as well as identified genes (Table S3) indicate that strain HdN1 employs the common reduction sequence (NO_3_^−^→ NO_2_^−^→ NO → N_2_O → N_2_), viz. is in principle able to readily reduce N_2_O. Also during growth with alkanes as organic substrates and NO_3_^−^ or NO_2_^−^ as electron acceptors, N_2_O must have been a regular intermediate because N_2_ rather than N_2_O was the end-product. However, N_2_O added alone did not allow growth with alkanes. An early reaction during alkane utilization must thus depend on a nitrogen–oxygen (N–O) species other than N_2_O. The early reaction could be the biochemically crucial activation of the alkane. The required N–O species cannot be NO_3_^−^, because growth with alkanes was also possible if NO_2_^−^ was added instead of NO_3_^−^. Hence, NO_2_^−^ or NO (or a so far unknown product from NO_2_^−^ reduction) may be essential for alkane activation. The basic hypothesis is depicted in Fig. 5. In further experiments, added NO (prepared from acidified NaNO_2_ and KI; Schreiber *et al*., 2008) turned out to be very toxic so that application of amounts theoretically sufficient to achieve measurable growth was not possible; NO at a partial pressure of *c*. 75 Pa [0.075% (v/v) in gas mixture of ambient pressure] in the headspace completely inhibited growth with NO_3_^−^. At a partial pressure of 50 Pa (0.05%) NO did not completely inhibit growth with NO_3_^−^, albeit growth was retarded. To test whether such still tolerated NO concentration is sufficient to initiate alkane degradation and in this way allow growth, 50 Pa NO was provided together with N_2_O (17 mmol l^−1^), the latter serving as main electron acceptor for anaerobic respiration. However, growth was not observed unless NO_3_^−^ was added. It thus remains elusive whether NO_2_^−^ or NO (or an unknown NO_2_^−^-derived species) is actually required to initiate alkane degradation.

**Fig. 6:**
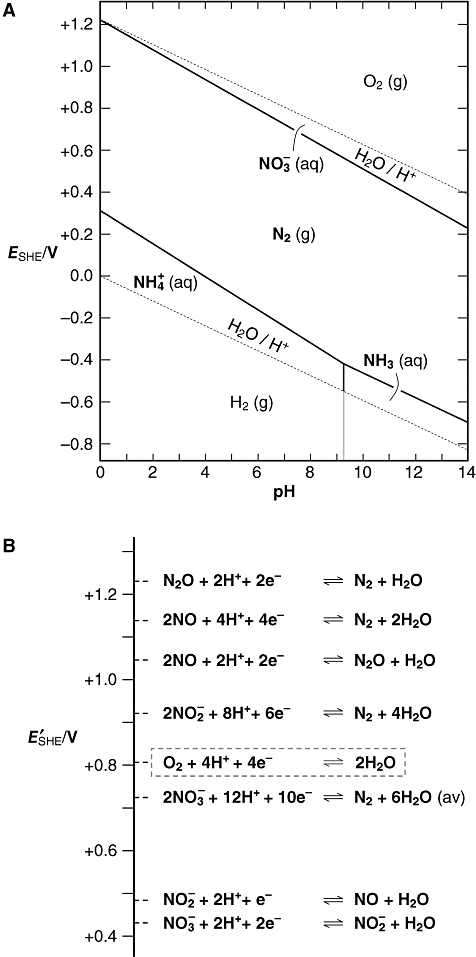
Some energetic aspects of N–O (and N–H) species. Graphs are for the following activities or fugacities: {NO_3_^−^}, {NO_2_^−^}, {NH_3_ (g)}, {NH_4_^+^} = 10^−2^; {N_2_ (g)} = 10^−0.1^ (78% in air); {O_2_ (g)} = 10^−0.7^ (21% in air); {H_2_} = 1; {NO (g)}, {N_2_O (g)} = 10^−4.3^ (approximately corresponding to dissolved concentrations monitored under natural conditions; Schreiber *et al*., 2009).A. *E*–pH (stability, Pourbaix) diagram of the system H–N–O. Only N_2_ and the lowest and highest oxidation states, NH_4_^+^, NH_3_ and NO_3_^−^, are thermodynamically stable. Other metabolically formed inorganic N-compounds are metastable (endergonic; e.g. Eqs 1 and 2) and can, in principle, spontaneously decompose (dismutate) into the species (including O_2_) depicted in the diagram. Endergonic N-compounds can be metabolically formed because they appear as co-products besides H_2_O (from reductive O elimination).B. Electrochemical half-reactions (including hypothetical ones) of N–O species and O_2_. If an endergonic N-compound does not react via dismutation (e.g. Eqs 1 and 2) but in an electrochemical half-reaction (yielding the same product as dismutation), this half-reaction has a higher redox potential than that of O_2_/2H_2_O (*E*°′ = +0.815 V). Again, this does not contradict the fact that NO_3_^−^ and NO_2_^−^ originate from a microbial oxidation process with O_2_ (see under A; also, the reaction sequence in nitrification is different: NH_4_^+^/NH_3_→ NH_2_OH → NO_2_^−^). Generally, the redox potentials (*E_i_*) of subsequent reduction steps (*i* = 1, 2, …, *m*) of an overall reduction with free intermediates are linked to an average redox potential (*E*_av_) according to (*n*_1_*E*_1_ + *n*_2_*E*_2_ + … + *n_m_E_m_*)/*n*_tot_ = *E*_av_; *n_i_* is the number of electrons involved in an individual step and *n*_tot_ the total number of electrons. *E*_av_ is connected to the total free energy change, Δ*G*_tot_, of the overall reduction with an electron-donating reaction of the redox potential *E*_don_ according to *E*_av_ = −Δ*G*_tot_/(*n*_tot_*F*) + *E*_don_, with *F* = 96 485 C mol^−1^ (explanation in Appendix S2). *E*_av_ of 2NO_3_^−^/N_2_ marks the borderline between the stability regions of NO_3_^−^ and N_2_ in the *E*–pH diagram (A). In a real metabolic process, a strong oxidant formed in a reduction sequence can only appear as free intermediate if its further reduction is enzymatically controlled and if unspecific reactions with reductants are slower or do not take place. Also, overall irreversibility is required, but this is naturally given (in an equilibrium system, redox pairs with different redox potential, e.g. NO_3_^−^/ NO_2_^−^ and NO_2_^−^/NO, cannot coexist).Calculations are based on standard free energy data (Garrels and Christ, 1965; Thauer *et al*., 1977). Derived standard redox potentials at pH = 7 (*E*°′/V): NO_3_^−^/NO_2_^−^, +0.431; 2NO_3_^−^/N_2_, +0.747 (av); NO_2_^−^/NO, +0.347; 2NO_2_^−^/N_2_, +0.958; 2NO/N_2_O, +1.172; 2NO/N_2_, +1.264; N_2_O/N_2_; +1.355.

From a thermodynamic point of view, an involvement of N–O species in alkane activation under anoxic conditions is an appealing hypothesis. N–O species other than NO_3_^−^ (Fig. 6A) are all metastable (Garrels and Christ, 1965; Thauer *et al*., 1977) and represent or can provide strong potential oxidants; this property may be enzymatically exploited to achieve alkane activation. An indirect use to form another reactive compound as well as a direct use of an N–O species can be envisaged.

One mode of indirect use of NO_2_^−^ and NO could be their dismutation (formally an ‘internal’ reduction of N and oxidation of O) leading to O_2_, according to the following equations:


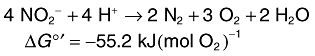
(1)


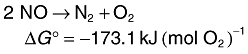
(2)

O_2_ could then be used for an alkane monooxygenase reaction (alkane hydroxylation). Also N_2_O can in principle lead to O_2_[2 N_2_O → 2 N_2_ + O_2_; Δ*G*° = −208.4 kJ (mol O_2_)^−1^], but the present results exclude its use for an initiation of alkane degradation. There is indeed evidence for O_2_ formation at very low concentration during NO_2_^−^ reduction in a methane-utilizing enrichment culture dominated by ‘*Candidatus* Methylomirabilis oxyfera’. The enrichment grew under exclusion of air and depended on NO_2_^−^ addition (Ettwig *et al*., 2010). ^18^O_2_ formation from N^18^O_2_^−^ (indirectly labelled through H_2_^18^O) became detectable upon specific inhibition of methane monooxygenase. NO dismutation (Eq. 2) was suggested as the underlying mechanism. Neither was NO_3_^−^ or N_2_O reduced, nor did the genome of the dominant bacterium harbour typical N_2_O-reductase genes. Results therefore suggested that NO dismutation was a main reaction during NO_2_^−^ reduction in ‘*Candidatus* M. oxyfera’. An earlier example of a metastable inorganic oxo-compound enabling biodegradative reactions through O_2_ formation is chlorite, an intermediate of microbial chlorate reduction (ClO_2_^−^→ Cl^−^ + O_2_; Δ*G°*′ = −148.4 kJ mol^−1^; Ginkel *et al*., 1996; Chakraborty and Coates, 2004; Tan *et al*., 2006; Weelink *et al*., 2008; Mehboob *et al*., 2009a,b;). The presently investigated alkane-degrading strain HdN1 differs metabolically from ‘M. oxyfera’ in several respects. Strain HdN1 does not utilize methane, grows with NO_3_^−^ and obviously involves the conventional reduction sequence via N_2_O to N_2_. If strain HdN1 would employ NO_2_^−^- or NO-derived O_2_, the demand per hydrocarbon molecule utilized would be much lower than in ‘*Candidatus* M. oxyfera’. Long-chain alkane activation would require only a minor withdrawal of NO_2_^−^ or NO from the respiratory path that mainly leads to N_2_ through N_2_O. For instance, *n*-hexadecanol resulting from oxygenation of *n*-hexadecane (C_15_H_31_CH_3_ + O_2_ + 2 [H]→ C_15_H_31_CH_2_OH + H_2_O) yields as many as 96 [H] (C_15_H_31_CH_2_OH + 31 H_2_O → 16 CO_2_ + 96 [H]) per substrate molecule. With 2 [H] consumed for activation, each oxygenation event thus leaves 94 [H] per C_16_H_34_ for respiratory energy conservation. In contrast, each oxygenation event in methane utilization provides only 4 [H] per CH_4_ for respiration. According to genomic data, strain HdN1 may form a di-iron monooxygenase, a P450-type monooxygenase and possibly a third type of monooxgenase (Table S4). Multiple monooxygenases are not uncommon in aerobic alkane degraders (Rojo, 2009).

O_2_ formation could not be detected so far in strain HdN1. We mixed a culture of strain HdN1 with a culture of luminous bacteria (isolated from herring using glycerol-peptone medium; Farmer and Hickman-Brenner, 2006) as sensitive O_2_ indicators (Chance *et al*., 1978); both cultures had been adapted to brackish water (180 mM NaCl and 20 mM MgSO_4_) medium. After extinction of luminescence due to oxygen consumption, neither addition of NO_3_^−^ nor of NO_2_^−^ or NO-saturated water caused the luminous reaction to resume (whereas air did immediately). Neither was oxygen detectable by means of an O_2_-microelectrode (lower detection limit, 1 µM; Revsbech, 1989) in cultures supplied with NO_2_^−^ or NO. Nevertheless, results do not rule out O_2_ as an intermediate. A very low production rate and effective scavenging by alkane monooxygenase and competing respiratory enzymes (if present under anoxic conditions) such as high-affinity *cbb*_3_-type oxidases (Pitcher and Watmough, 2004; predicted for strain HdN1; Table S5) could maintain the O_2_ concentration below detection level. Also, the produced alcohol may be consumed effectively by the subsequent reaction. Only upon sudden exposure to air, the anaerobically grown cells accumulated detectable *n*-hexadecanol (see above).

The slight differences between the growth tests under oxic and anoxic conditions with alkanes of various chain lengths (see growth tests with different electron acceptors) do not necessarily contradict the hypothesis that monooxygenases are used under oxic as well as under anoxic cultivation conditions for alkane activation. There might be slight differences with respect to chain length specificity between the monooxygenase(s) formed in aerobic and denitrifying cultures.

Still, also other modes of an indirect use of N–O species for alkane activation can be envisaged. For instance, they may serve as high-potential (strongly oxidizing) electron acceptors (half reactions in Fig. 6B) to generate by electron withdrawal an oxidized, active (reactive) state of a factor or an enzyme site involved in alkane activation.

Another hypothesis would be the direct involvement of an N–O species in the activation reaction of the alkane. Such direct biochemical linkage of the disintegration of an N–O species to C−H bond cleavage and formation of a functionalized product from an alkane would probably require an intricate mechanism.

### Concluding remarks

In conclusion, results suggest a mechanistic alternative to the fumarate-dependent reaction for anaerobic alkane activation. Also a sulfate-reducing bacterium, strain Hxd3, metabolized long-chain *n*-alkanes obviously via an initial reaction different from that in other anaerobic alkane degraders (Aeckersberg *et al*., 1998; So *et al*., 2003; Callaghan *et al*., 2006). This raises the question whether nitrate-reducing strain HdN1 and sulfate-reducing strain Hxd3 employ basically the same reaction or different reactions to initiate alkane degradation. If they would employ essentially the same fumarate-independent activation reaction, strain HdN1 cannot employ an N–O species or derived O_2_ directly in the mechanism because they are excluded in strain Hxd3; the sulfate reducer was grown without nitrate. Also other ways to generate O_2_ are essentially excluded in sulfate reducers; sulfate and its metabolites are all thermodynamically very stable and represent very weak oxidants. Hence, alkane activation by the same basic mechanism in strains HdN1 and Hxd3 would imply that the denitrifier uses an N–O species or O_2_ only indirectly to generate an alkane-activating factor, whereas the sulfate reducer would generate the same type of factor in a different manner. If strains HdN1 and Hxd3 use different mechanisms for alkane activation, which is more appealing to assume, the reaction in strain HdN1 would represent a third type of alkane activation under anoxic conditions, besides the fumarate-dependent mechanism and the speculative mechanism in strain Hxd3. More refined physiological experiments (preceded by an improved method for harvesting the buoyant cells associated with alkane) are needed to provide further hints as to the alkane activation mechanism in strain HdN1, with consideration of its apparently diverse monooxygenases.

Finally, the present results as well as the oxidation of methane with NO_2_^−^ (Ettwig *et al*., 2010) indicate that NO_3_^−^ or NO_2_^−^ (either from NO_3_^−^ reduction or directly from NH_4_^+^ oxidation) should not be considered merely as electron acceptors for anaerobic respiration. An intermediate formed during NO_3_^−^ or NO_2_^−^ reduction may provide or function as a co-reactant for the biochemical activation of various hydrocarbons or even of other chemically unreactive compounds. NO_3_^−^ or NO_2_^−^ in anoxic habitats could, in principle, promote or enable the degradation of certain organic fractions which tend to be refractory under conditions of sulfate reduction or methanogenesis.

## Supporting information

**Fig. S1.** Relationship of the assumed catalytic (large) subunit (MasD) of the *n*-alkane-activating enzyme in strain OcN1 to other enzymes activating hydrocarbons by addition to fumarate.

**Table S1.** General genome features of strain HdN1.

**Table S2.** Genome-based search for genes (*bssA* and homologues) that may encode fumarate-dependent glycyl radical enzymes for anaerobic alkane activation.

**Table S3.** Genome-based prediction of enzymes for the denitrification pathway in strain HdN1.

**Table S4.** Genome-based prediction of alkane monooxygenases (alkane hydroxylases) in strain HdN1.

**Table S5.** Genome-based prediction of *cbb*_3_-type oxidases (oxidases with high O_2_ affinity) in strain HdN1.

**Appendix S1.** Cultivation of strains, physiological experiments.

**Appendix S2.** Formal derivation of the average redox potential.
