# Supplementary material for: Alkane degradation under anoxic conditions by a nitrate-reducing bacterium with possible involvement of the electron acceptor in substrate activation
Source: Environ Microbiol Rep. 2011 Feb;3(1):125–35. doi: 10.1111/j.1758-2229.2010.00198.x (PMC3151549; doi:10.1111/j.1758-2229.2010.00198.x)
Supplement: Supplementary file 1 [file emi40003-0125-SD1.pdf]

Zedelius *et al*.

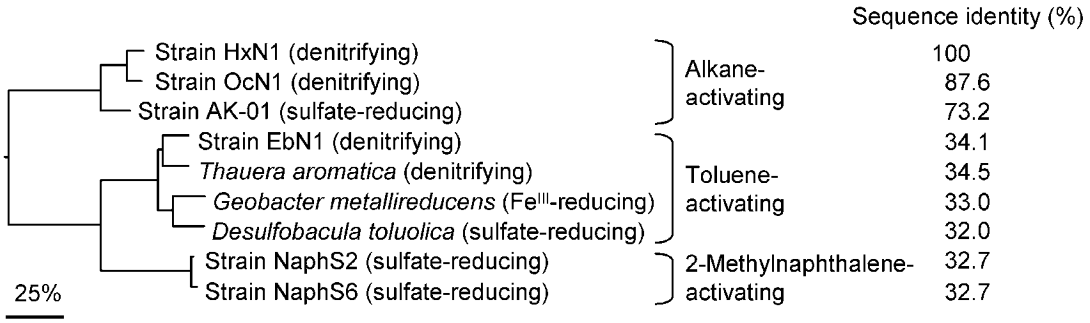

Fig. S1. Relationship of the assumed catalytic (large) subunit (MasD) of the *n*-alkane-activating enzyme in strain OcN1 to other enzymes activating hydrocarbons via addition to fumarate. Bar, 25% amino acid exchange.
Accession numbers: HxN1, CAO03074.1; OcN1, FN675935; AK-01, ABH11460.1; EbN1, YP_158060.1; *T. aromatica*, AAC38454.1; *G. metallireducens*, AAM34597.1; *D. toluolica*, ABM92935.1; NaphS2, CAO72219.1; NaphS6, CAO72222.1.
