# Supplementary material for: Alkane degradation under anoxic conditions by a nitrate-reducing bacterium with possible involvement of the electron acceptor in substrate activation
Source: Environ Microbiol Rep. 2011 Feb;3(1):125–35. doi: 10.1111/j.1758-2229.2010.00198.x (PMC3151549; doi:10.1111/j.1758-2229.2010.00198.x)
Supplement: Supplementary file 2 [file emi40003-0125-SD2.pdf]

Table S1. General genome[a] features of strain HdN1.

Size (bp)	4,587,455	
G + C content (mol%)	53.26	
Stable RNAs		
rRNAs	9	(3 operons)
tRNAs	47	
Protein-coding sequences (CDS)[b]	3,763	
Coding (%)	89.2	
Average length (bp)	1,088	
Pseudo genes	45	

[a] Accession no. FP929140
[b] Without "pseudo"-qualifier
