# Supplementary material for: Alkane degradation under anoxic conditions by a nitrate-reducing bacterium with possible involvement of the electron acceptor in substrate activation
Source: Environ Microbiol Rep. 2011 Feb;3(1):125–35. doi: 10.1111/j.1758-2229.2010.00198.x (PMC3151549; doi:10.1111/j.1758-2229.2010.00198.x)
Supplement: Supplementary file 3 [file emi40003-0125-SD3.pdf]

Zedelius *et al*.

Table S2. Genome-based TBLASTN-search for genes (*bssA* and homologues) that may encode fumarate-dependent glycyl radical enzymes for anaerobic alkane activation.

**Query**				**Best TBLASTN-hit in genome of strain HdN1**			
**Gene name**	**Acc. no.**	**Amino acids**	**Predicted function, organism**	**Position**	**E-value**	**Identities**	**Positives**
bssA	CAI07159	861	α-Subunit, benzylsuccinate synthase, denitrifying Betaproteobacterium strain EbN1	762,138 – 761,950	1.2	20/70 (29%)	30/70 (43%)
masD	CAO03074	839	α-Subunit, (1-methylalkyl)succinate synthase, denitrifying Betaproteobacterium strain HxN1	1,510,228 – 1,510,115	0.3	16/38 (42%)	24/38 (63%)
nmsA	CAO72219	828	α-Subunit, (2-naphthylmethyl)succinate synthase, sulfate-reducing Deltaproteobact. strain NaphS2	1,591,168 – 1,591,049	1.1	15/40 (38%)	20/40 (50%)
dhaB1	CAJ67970	790	Glycerol dehydratase, *Clostridium difficile* 630	235,877 – 235,695	4.1	22/73 (30%)	31/73 (42%)
pflB	P09373	760	Pyruvate formate lyase, *Escherichia coli* K12	2,685,988 – 2,686,473	0.27	41/170 (24%)	74/170 (44%)
nrdD	P28903	712	Anaerobic ribonucleotide reductase, *Escherichia coli* K12	1,500,318 – 1,500,040	2.1	25/95 (26%)	41/95 (43%)

TBLASTN was performed as described by Altschul, S.F., Madden, T.L., Schaffer, A.A., Zhang, J., Zhang, Z., Miller, W., and Lipman, D.J. (1997) Gapped BLAST and PSI-BLAST: a new generation of protein database search programs. *Nucleic Acids Res* **25:** 3389–3402.
