# Supplementary material for: Alkane degradation under anoxic conditions by a nitrate-reducing bacterium with possible involvement of the electron acceptor in substrate activation
Source: Environ Microbiol Rep. 2011 Feb;3(1):125–35. doi: 10.1111/j.1758-2229.2010.00198.x (PMC3151549; doi:10.1111/j.1758-2229.2010.00198.x)
Supplement: Supplementary file 4 [file emi40003-0125-SD4.pdf]

Table S3. Genome-based prediction of enzymes for the common denitrification pathway in strain HdN1.

Predicted gene (product) of strain HdN1					BLASTP hit used for annotation			
Gene name	Identifier	Start (bp)	Amino acids	Predicted function	Gene	Organism [a]	E-value	Acc. no.
**Cluster I** (1,639,227 – 1,652,317)								
narL	HDN1F_13190	1,639,865	213	Nitrate/nitrite 2-component response regulator	*narL*	Pseae	1e-59	O54039
narX	HDN1F_13210	1,641,736	624	Nitrate/nitrite 2-component sensor kinase	*narX*	Pseae	1e-94	O54040
narK1	HDN1F_13220	1,642,206	453	Nitrate/proton symporter	*narK1*	Pseae	7e-98	O54041
narK2	HDN1F_13230	1,643,621	556	Nitrate/nitrite antiporter	*narK2*	Azose	0.0	Q5NYZ3
narG	HDN1F_13240	1,645,636	1250	Nitrate reductase, alpha subunit	*narG*	Azose	0.0	Q5NYZ4
narH	HDN1F_13250	1,649,402	510	Nitrate reductase, beta subunit	*narH*	Azose	0.0	Q5NYZ5
narJ	HDN1F_13260	1,650,937	230	Nitrate reductase, delta subunit	*narJ*	Pseae	3e-42	O54045
narI	HDN1F_13270	1,651,640	226	Nitrate reductase, gamma subunit	*narI*	Pseae	3e-72	O54046
**Cluster II** (4,472,792 – 4,479,005)								
nirM	HDN1F_37060	4,472,792	104	Cytochrome *c*, monohaem	*nirM*	Pseae	3e-22	P00099
nirS	HDN1F_37070	4,474,578	585	Dissimilatory cytochrome cd_1 nitrite reductase precursor	*nirS*	Azose	0.0	Q5P7W3
norD	HDN1F_37080	4,476,628	631	Predicted nitric oxide reductase activation protein	*norD*	Pseae	1e-120	O51484
norQ	HDN1F_37090	4,477,453	273	Putative chaperone required for maturation of nitric oxide reductase	*norQ*	Azose	9e-95	Q5P8Y1
norB	HDN1F_37100	4,478,983	470	Nitric oxide reductase subunit B	*norB*	Azose	0.0	Q5P8Y4
norC	HDN1F_37110	4,479,430	142	Nitric oxide reductase subunit C	*norC*	Azose	2e-59	Q5P8Y5
**Cluster III** (4,522,585 – 4,528,867)								
nosL	HDN1F_37530	4,522,585	129	Predicted lipoprotein involved in nitrous oxide reduction (COG4314)	-	-	-	-
nosY	HDN1F_37540	4,523,550	271	Probable transmembrane protein	*nosY*	Azose	2e-74	Q5NZ06
nosF	HDN1F_37550	4,524,502	308	Putative ATP-binding protein	*nosF*	Azose	6e-94	Q5NZ05
nosD	HDN1F_37560	4,525,925	448	Nitrous oxidase accessory protein precursor	*nosD*	Azose	1e-149	Q5NZ04
nosR	HDN1F_37570	4,528,549	875	Domain, 4Fe-4S ferredoxin, iron-sulfur binding, FMN-binding	-	-	-	-
nosZ	HDN1F_37580	4,530,828	654	Nitrous oxide reductase	*nosZ*	Azose	0.0	Q5NZ01

[a] Abbreviations for organisms: Pseae, *Pseudomonas aeruginosa*; Azose, *Azoarcus*-related denitrifying Betaproteobacterium strain EbN1.
