# Supplementary material for: Alkane degradation under anoxic conditions by a nitrate-reducing bacterium with possible involvement of the electron acceptor in substrate activation
Source: Environ Microbiol Rep. 2011 Feb;3(1):125–35. doi: 10.1111/j.1758-2229.2010.00198.x (PMC3151549; doi:10.1111/j.1758-2229.2010.00198.x)
Supplement: Supplementary file 5 [file emi40003-0125-SD5.pdf]

Table S4. Genome-based prediction of alkane monooxygenases (alkane hydroxylases) in strain HdN1.

Predicted gene (product) of strain HdN1					BLASTP hit used for annotation			
**Identifier**	**Start (bp)**	**Amino acids**	**Predicted function**	**InterPro [a]**	**Gene**	**Organism [b]**	**E-value**	**Acc. no.**
Cyt P450 monooxygenase *(accessory proteins[c] in grey)*								
HDN1F_17550	2,213,848	106	Ferredoxin	IPR001041		Alcbo	2e-44	Q5ca10
HDN1F_17560	2,214,183	471	Cytochrom P450 alkane hydroxylase	IPR001128	*ahpG2*	Alcbo	0	Q5K133
HDN1F_17570	2,215,803	410	Ferredoxin reductase	IPR001327		Alcbo	0	Q5ca08
Alkane-1-monooxygenase *(di-iron enzyme)*								
HDN1F_04180	517,699	314	Transcriptional regulator, AlkR	IPR000005	*alkR*	Acica	4e-71	Q9xdp8
HDN1F_04190	517,997	423	Alkane 1-monoxygenase, AlkM (di-iron)	IPR012145		Aciba	1e-138	ZP_05827357
Alkane-1-monooxygenase *(flavin enzyme)*								
HDN1F_14540	1,814,074	505	Alkane 1-monoxygenase (flavin)	IPR000960		Alcbo	9e-167	YP_692002
HDN1F_17470	2,204,606	500	Alkane 1-monoxygenase (flavin)	IPR000960		Alcbo	0	YP_692002
HDN1F_17580	2,218,527	512	Alkane 1-monoxygenase (flavin)	IPR000960		Alcbo	0	YP_691914
HDN1F_30030	3,708,484	495	Alkane 1-monoxygenase (flavin)	IPR000960		Alcbo	2e-178	YP_692002
HDN1F_36680	4,423,219	511	Alkane 1-monoxygenase (flavin)	IPR000960		Alcbo	3e-159	YP_691914

[a] IPR001041: domain, ferredoxin; IPR001128: family, cytochrome P450; IPR001327: domain, FAD-dependent pyridine nucleotide-disulfide oxidoreductase; IPR000005: domain, helix-turn-helix, AraC type; IPR012145: family, alkane 1-monooxygenase; IPR000960: family, flavin-containing monooxygenase FMO.
[b] Abbreviations for organisms: Aciba, *Acinetobacter baumannii*; Acica, *Acinetobacter calcoaceticus*; Alcbo, *Alcanivorax borkumensis*.
[c] Genes for accessory proteins in direct proxemity to the predicted oxygenase-related genes.
