# Supplementary material for: Alkane degradation under anoxic conditions by a nitrate-reducing bacterium with possible involvement of the electron acceptor in substrate activation
Source: Environ Microbiol Rep. 2011 Feb;3(1):125–35. doi: 10.1111/j.1758-2229.2010.00198.x (PMC3151549; doi:10.1111/j.1758-2229.2010.00198.x)
Supplement: Supplementary file 6 [file emi40003-0125-SD6.pdf]

Table S5. Genome-based prediction of *cbb*$_3$-type oxidases (oxidases with high O_2-affinity) in strain HdN1.

Predicted gene (product) of strain HdN1						BLASTP hit used for annotation			
**Gene name**	**Identifier**	**Start (bp)**	**Amino acids**	**Predicted function**	**InterPro [a] COG [b,c]**	**Gene**	**Organism [d]**	**E-value**	**Acc. no.**
ccoP	HDN1F_11960	1,483,054	298	Cytochrome *c* oxidase *cbb*$_3$-type, subunit III	IPR004678 COG2010	*ccoP*	Azose	1e-65	Q5P0X3
ccoQ	HDN1F_11970	1,483,947	76	Probable cytochrome *c* oxidase, subunit CcoQ	IPR008621 COG4736	*ccoQ*	Azose	1e-04	Q5P0X4
ccoO	HDN1F_11980	1,484,180	207	Cytochrome *c* oxidase, *cbb*$_3$-type, subunit II	IPR003468 COG2993	-	-	-	-
ccoN	HDN1F_11990	1,484,818	480	Cytochrome *c* oxidase, *cbb*$_3$-type, subunit I	IPR004677 COG3278	-	-	-	-
ccoN	HDN1F_27210	3,357,106	498	Cytochrome-*cbb*$_3$ oxidase, subunit I	IPR004677	*ccoN*	Azose	1e-145	Q5P0X6
ccoO	HDN1F_27220	3,358,613	216	Cytochrome-*cbb*$_3$ oxidase, subunit I	IPR003468 COG2993	*ccoO*	Azose	3e-54	Q5P0X5
ccoQ	HDN1F_27230	3,359,260	63	Cytochrome *c* oxidase subunit	IPR008621	-	-	-	-
ccoP	HDN1F_27240	3,359,444	307	Cytochrome-*cbb*$_3$ oxidase, subunit III	IPR004678 COG2010	*ccoP*	Azose	2e-48	Q5P0X3

[a] IPR003468: family, cytochrome *c* oxidase, mono-heme subunit; IPR004677: family, cytochrome *c* oxidase *cbb*$_3$-type, subunit I; IPR004678: family, cytochrome *c* oxidase *cbb*$_3$-type, subunit III; IPR008621: family, *cbb*$_3$-type cytochrome oxidase, cytochrome *c* subunit.
[b] COG, cluster of orthologous groups.
[c] COG2010: CccA, cytochrome *c*, mono- and diheme variants; COG2993: CcoO, *cbb*$_3$-type cytochrome oxidase, cytochrome *c* subunit; COG3278: CcoN, *cbb*$_3$-type cytochrome oxidase, subunit I; COG4736: *cbb*$_3$-type cytochrome oxidase, subunit 3.
[d] Abbreviation for organism: Azose, *Azoarcus*-related denitrifying Betaproteobacterium strain EbN1.
