# Supplementary material for: Alkane degradation under anoxic conditions by a nitrate-reducing bacterium with possible involvement of the electron acceptor in substrate activation
Source: Environ Microbiol Rep. 2011 Feb;3(1):125–35. doi: 10.1111/j.1758-2229.2010.00198.x (PMC3151549; doi:10.1111/j.1758-2229.2010.00198.x)
Supplement: Supplementary file 7 [file emi40003-0125-SD7.pdf]

Appendix S1

Materials and methods

Origin of strains and regular cultivation

The denitrifying strain HdN1 was originally enriched with refined (aliphatic) mineral oil from freshwater mud and isolated with hexadecane (Ehrenreich *et al*., 2000). Strains HxN1 and OcN1 were enriched and isolated in the same study with hexane and octane, respectively. Cultures were maintained in the laboratory by transfer every 3 to 5 months, growth for approximately one week, and storage at 4 °C in the dark.

Anaerobic cultures were routinely grown in butyl rubber-sealed tubes (20 ml) or bottles (110 ml) with 10 ml and 80 ml anoxic medium, respectively (details in Widdel and Bak, 1992; Rabus and Widdel, 1995; Ehrenreich *et al*., 2000). Briefly, the medium for routine cultivation was prepared with 30 mM $NaHCO_3$ and a low phosphate concentration (3.7 mM). The reductant was ascorbate (4 mM) added from a filter-sterilized stock solution (prepared anaerobically from ascorbic acid and NaOH). Ascorbate did not serve as a substrate for growth. Usually, 10 mM $NaNO_3$ was added. The medium was overlaid with pure sterile (autoclaved under N_2) hexadecane (1.5 to 5 µl per ml medium, corresponding to 5.1 – 17.1 mmol l^{-1}).

Purity control

Cultures of strains grown with alkanes and other organic substrates were regularly checked by phase contrast microscopy. To verify absence of contaminants, cultures were also transferred to diagnostic liquid medium containing peptone (5 g l^{-1}) or yeast extract (5 g l^{-1}) and incubated anaerobically with NO_3^- or aerobically under air. In addition, cultures were streaked on agar plates prepared with fumarate (5 mM) or valerate (4 mM) and yeast extract (2.5 g l^{-1}) and incubated under air with 3% CO_2. Single, separate colonies were examined microscopically and tested for anaerobic growth with tetradecane.

Growth and growth tests with various electron donors and electron acceptors

The alkanes dodecane, tridecane and tetradecane were added like hexadecane (see above) as pure sterile liquids. Alkanes with shorter carbon chains, octanol and decanol were dissolved (pentane through octane, 5%; octanol, decanol, 1%; otherwise 20%) in 2,2,4,4,6,8,8-heptamethylnonane as an inert and non-toxic carrier phase. The added volume of the alkane solutions was usually 50 µl per ml culture volume. Solid alkanes and solid long-chain alcohols were first added to empty culture tubes. Upon mild heating, the molten compounds were allowed to solidify while the tube was rotated so as to cover the glass wall with a thin layer. Then the tubes were flushed with oxygen-free gas while medium was added. Concentrations of monocarboxylic acid salts (in mM): formate, acetate, 10; propionate, butyrate, 5; valerate, 4; caproate, 3, heptanoate, 2; octanoate, 2; nonanoate, decanoate, dodecanoate, 1; tetradecanoate, 2, palmitate, 1.5; stearate, 1.5; long-chain fatty acid salts were added as molten solutions (Widdel and Bak, 1992). Ethanol was added at 10 mM.

Aerobic cultures were grown in sealed serum bottles with medium under air; the aqueous phase was one fifth or less of the bottle volume. The oxic medium without ascorbate contained only 2 mM $NaHCO_3$, while the headspace contained 1% CO_2.

Production of N_2, N_2O or CO_2, or consumption of N_2O was measured in cultures with more phosphate (KH_2PO_4, 8 mM, K_2HPO_4, 30 mM; pH ≈ 7.1) and without $NaHCO_3$ under a head space of pure argon.

Commercial N_2O (99.5%) for growth tests was incubated before application for at least two days at an overpressure of 100 kPa in a butyl-rubber sealed 165-ml serum bottle containing 5 ml of alkaline (pH 11) ascorbate (1 M) for scavenging traces of O_2. A volume of 0.61 ml was injected per ml of liquid culture volume (ca. 25 mmol l^{-1}).

NO was prepared from acidified $NaNO_2$ and KI as described previously (Schreiber *et al*., 2008) and stored in butyl rubber-sealed 5-ml glass tubes. Small volumes were withdrawn by means of a microliter syringe and injected into culture vials with large anoxic headspace (165 ml) and small aqueous volumes (10 ml) so as to achieve partial pressures of 50 – 100 Pa (0.05 – 0.1% in an atmosphere of ambient pressure).

Sequence analysis of 16S rRNA genes

Extraction of genomic DNA from strains HdN1, HxN1 and OcN1, amplification of 16S rRNA genes by PCR, and purification of PCR products were carried out using established procedures (Rainey *et al.,* 1996). Purified PCR products were sequenced using the Taq DyeDeoxy Terminator Cycle Sequencing Kit (Applied Biosystems, Foster City, Calif., USA) and the 373S instrument (Applied Biosystems).

Sequences were aligned with those of the SILVA SSU database (www.arb-silva.de, August 2009; Pruesse *et al*., 2007). The phylogenetic tree was constructed by means of the RAxML (maximum likelihood) program (7.0.3, release March 2008; Stamatakis *et al.*, 2005) of the ARB package (Ludwig *et al*., 2004) by applying position-variability and termini filters. Bootstrap values were obtained after 1000 resamplings. Only high quality sequences (>1200 bp) were used for tree construction. The 16S rRNA sequences of strains HdN1, HxN1 and OcN1 are available from the EMBL database under accession numbers Y17827, Y17826 and Y17828 respectively.

Cell hybridization assay

Fluorescent "*in-situ*" (whole-cell) hybridizations (FISH) for purity controls was carried out as described (Musat *et al.*, 2008) using the newly designed oligonucleotide probe HdN1_112 (5'-TTCCTGCGCTATCCTCAC-3', 60% formamide) which is specific for strain HdN1. The highest stringency yielding hybridization was obtained with 60% formamide. None of the 16S rRNA sequences in the SILVA SSU database (www.arb-silva.de, release August 2009; Pruesse *et al*., 2007) exhibited less than four mismatches.

Genomic analysis

DNA was isolated with the Genomic DNA kit (Qiagen, Hildesheim, Germany) according to the manufacturer's instructions and sonified. Shotgun fragments were ligated into the pUC/SmaI vector (Fermentas, St. Leon-Rot, Germany) and electroporated into *Escherichia coli* strain K12 substrain DH10B. Insert sizes were between 1.5 and 2.5 kb. In addition, a fosmid library (CopyControl™ Fosmid Library Production Kit, Epicentre,

Madison, U.S.A.) was constructed for data finishing and confirmation of the assembly. Recombinant DNA was sequenced using the ABI3730XL instrument (ABI, Darmstadt, Germany). Gaps and regions of insufficient quality were completed by resequencing and primer walking. Sequences were assembled with PhredPhrap (http://www.phrap.org). The Consed package (Gordon, 2003) was used for final sequence editing. An 11-fold sequencing coverage was reached, resulting in a sequence quality of less than 1 error per 100,000 bases (average). Genes that may encode glycyl radical enzymes, enzymes of the known denitrification pathway, oxygenases, and *cbb*$_3$-type oxidases were searched for using the BLASTP-program (Altschul *et al*., 1997) and the HTGA-system (Rabus *et al*., 2002).

Chemical analyses

Nitrate and nitrite were measured by high-performance liquid chromatography (Sykam, Gliching/Munich, Germany) as described (Rabus und Widdel, 1995).

N_2, CO_2 and N_2O were analyzed in the culture headspace (see above) by gas chromatography using a GC-8A instrument (Shimadzu, Duisburg, Germany) equipped with a thermal conductivity detector. The gases were separated on a CP PoraPLOT Q (3 mm × 2 mm; Agilent, Waldbronn, Germany) or HP-PLOT MoleSieve (0.53 mm × 0.32 mm; Agilent, Waldbronn, Germany) column at 40°C using argon as carrier gas at a flow rate of 15.0 ml min^{-1}. Headspace samples were withdrawn with a gas-tight syringe equipped with a gas valve.

Organic acids were extracted from heat-inactivated (85 °C) and acidified cultures with dichloromethane, methylated, and analyzed by gas chromatography-mass spectrometry as described (Rabus *et al*., 2001). Long-chain alcohols were extracted with diethylether. After evaporation the residue was silylated with N,O-bis(trimethylsilyl)acetamide (Supelco, product specification T496017A) and analyzed by gas chromatography-mass spectrometry using a GCQ instrument (ThermoQuest Finnigan, Bremen, Germany) equipped with an Optima-5 column (Macherey-Nagel, Düren, Germany); the carrier gas was helium.

References

Altschul, S.F., Madden, T.L., Schäffer, A.A., Zhang, J., Zhang, Z., Miller, W., and Lipman, D.J. (1997) Gapped BLAST and PSI-BLAST: a new generation of protein database search programs. *Nucleic Acids Res* **25:** 3389–3402.

Ehrenreich, P., Behrends, A., Harder, J., and Widdel, F. (2000) Anaerobic oxidation of alkanes by newly isolated denitrifying bacteria. *Arch Microbiol* **173:** 58–64. (Erratum: *Arch Microbiol* **173:** 232).

Gordon, D. (2003) Viewing and editing assembled sequences using Consed. Current Protocols in Bioinformatics. Chapter 11, Unit 11 12.

Ludwig, W., Strunk, O., Westram, R., Richter, L., Meier, H., Yadhukumar et al. (2004) ARB: A software environment for sequence data. *Nucleic Acids Res* **32**: 1363–1371.

Musat, F., and Widdel, F. (2008) Anaerobic degradation of benzene by a marine sulfate-reducing enrichment culture, and cell hybridization of the dominant phylotype. *Env Microbiol* **10**: 10–19.

Pruesse, E., Quast, C., Knittel, K., Fuchs, B.M., Ludwig, W.G., Peplies, J., and Glöckner, F.O. (2007) SILVA: a comprehensive online resource for quality checked and aligned ribosomal RNA sequence data compatible with ARB. *Nucleic Acids Res* **35**: 7188–7196.

Rabus, R., Kube, M., Beck, A., Widdel, F., and Reinhardt, R. (2002) Genes involved in the anaerobic degradation of ethylbenzene in a denitrifying bacterium, strain EbN1. *Arch Microbiol* **178:** 506–516.

Rabus, R., Wilkes, H., Behrends, A., Armstroff, A., Fischer, T., Pierik, A.J., and Widdel, F. (2001) Anaerobic initial reaction of *n*-alkanes: evidence for (1-methylpentyl)succinate as initial product and for involvement of an organic radical in the metabolism of *n*-hexane in a denitrifying bacterium. *J Bacteriol* **183:** 1707–1715.

Rabus, R., and Widdel, F. (1995) Anaerobic degradation of ethylbenzene and other aromatic-hydrocarbons by new denitrifying bacteria. *Arch Microbiol* **63**: 96–103.

Rainey, F.A., WardRainey, N., Kroppenstedt, R.M., and Stackebrandt, E. (1996) The genus *Nocardiopsis* represents a phylogenetically coherent taxon and a distinct actinomycete lineage: Proposal of Nocardiopsaceae fam nov. *Int J Syst Bacteriol* **46**: 1088–1092.

Schreiber, F., Polerecky, L., de Beer, D. (2008) Nitric oxide microsensor for high spatial resolution measurements in biofilms and sediments. *Anal Chem* **80:** 1152–1158.

Stamatakis, A., Ludwig, T., and Meier, H. (2005) RAxML-III: a fast program for maximum likelihood-based inference of large phylogenetic trees. *Bioinformatics* **21**: 456–463.

Widdel, F., and Bak, F. (1992) Gram-negative mesophilic sulfate-reducing bacteria. In *The prokaryotes*. Balows, A., Trüper, H.G., Dworkin, M., Harder, W., and Schleifer, K.-H. (eds). New York: Springer, pp. 3352–3378.
