# Supplementary material for: Alkane degradation under anoxic conditions by a nitrate-reducing bacterium with possible involvement of the electron acceptor in substrate activation
Source: Environ Microbiol Rep. 2011 Feb;3(1):125–35. doi: 10.1111/j.1758-2229.2010.00198.x (PMC3151549; doi:10.1111/j.1758-2229.2010.00198.x)
Supplement: Supplementary file 8 [file emi40003-0125-SD8.pdf]

Zedelius *et al*.

Appendix S2

Redox potentials of subsequent reduction steps, and average redox potential

If an electron acceptor A_{ox} is reduced stepwise to A_{red} via m subsequent steps involving free intermediates (I_i), the reaction can be written as follows (n_i, number of electrons):

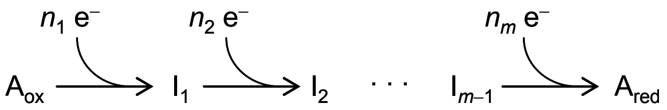

With an electron donor of a given redox potential, E_{don}, and the redox potential E_i of a reduction step (definition of which is originally based on a reversible reaction), the free energy change of an individual step is $-\Delta G_i = n_i\, F\, \Delta E_i = n_i\, F\, (E_i - E_{don})$; F = 96,485 C mol^{-1} (Faraday constant). The free energy change of the total reduction reaction is

$$-\Delta G_{tot} \;=\; n_1\, F\, (E_1 - E_{don}) + n_2\, F\, (E_2 - E_{don}) + \ldots + n_m\, F\, (E_m - E_{don}) \qquad (1)$$

$$=\; F\, [(n_1 E_1 + n_2 E_2 + \ldots + n_m E_m) - (n_1 + n_2 + \ldots + n_m) E_{don}] \qquad (2)$$

The sum $n_1 + n_2 + \ldots + n_{\mathrm{i}}$ is the total number of electrons, n_{tot}, so that

$$-\Delta G_{tot} \;=\; F\, [(n_1 E_1 + n_2 E_2 + \ldots + n_m E_m) - n_{tot} E_{don}]\ . \qquad (3)$$

Rearrangement leads to

$$\frac{-\Delta G_{tot}}{n_{tot}\, F} + E_{don} \;=\; \frac{n_1 E_1 + n_2 E_2 + \ldots + n_m E_m}{n_{tot}}\ . \qquad (4)$$

The term $-\Delta G_{tot}/(n_{tot}\, F)$ expresses a redox potential difference. Because it includes only parameters referring to the total reaction, it can be defined as the average redox potential difference, ΔE_{av}, of the total reaction:

$$\frac{-\Delta G_{tot}}{n_{tot}\, F} = \Delta E_{av}\, . \qquad (5)$$

Because ΔE_{av} is relative to the redox potential of the donor reaction, (5) necessarily defines also an average redox potential by $\Delta E_{av} = E_{av} - E_{don}$. We can thus write (4) as

$$(E_{av} - E_{don}) + E_{don} \;=\; E_{av} \;=\; \frac{n_1 E_1 + n_2 E_2 + \ldots + n_m E_m}{n_{tot}} \qquad (6)$$
